# Adaptive and innovative Radiation Treatment FOR improving Cancer treatment outcomE (ARTFORCE); a randomized controlled phase II trial for individualized treatment of head and neck cancer

**DOI:** 10.1186/1471-2407-13-84

**Published:** 2013-02-22

**Authors:** Jolien Heukelom, Olga Hamming, Harry Bartelink, Frank Hoebers, Jordi Giralt, Teresa Herlestam, Marcel Verheij, Michiel van den Brekel, Wouter Vogel, Nick Slevin, Eric Deutsch, Jan-Jakob Sonke, Philippe Lambin, Coen Rasch

**Affiliations:** 1 Department of radiation oncology, Netherlands Cancer Institute, Amsterdam, The Netherlands; 2 Department of radiation oncology, Maastro Clinics, Maastricht, The Netherlands; 3 Department of radiation oncology, Val d’Hebron Hospital, Barcelona, Spain; 4 Department of radiation oncology, Karolinska Hospital, Stockholm, Sweden; 5 Department of ear, nose and throat oncology and head and neck surgery, Netherlands Cancer Institute, Amsterdam, The Netherlands; 6 Department of nuclear medicine, Netherlands Cancer Institute, Amsterdam, The Netherlands; 7 Department of radiation oncology, Christie Hospital, Manchester, United Kingdom; 8 Department of radiation oncology, Institut Gustave Roussy, Paris, France; 9 Department of radiation oncology, Amsterdam Medical Centre, Amsterdam, The Netherlands

**Keywords:** Head and neck, Squamous cell carcinoma, Adaptive radiotherapy, Dose painting, Zirconium, Cetuximab, Cisplatin

## Abstract

**Background:**

Failure of locoregional control is the main cause of recurrence in advanced head and neck cancer. This multi-center trial aims to improve outcome in two ways. Firstly, by redistribution of the radiation dose to the metabolically most FDG-PET avid part of the tumour. Hereby, a biologically more effective dose distribution might be achieved while simultaneously sparing normal tissues. Secondly, by improving patient selection. Both cisplatin and Epidermal Growth Factor Receptor (EGFR) antibodies like Cetuximab in combination with Radiotherapy (RT) are effective in enhancing tumour response. However, it is unknown which patients will benefit from either agent in combination with irradiation. We will analyze the predictive value of biological markers and ^89^Zr-Cetuximab uptake for treatment outcome of chemoradiation with Cetuximab or cisplatin to improve patient selection.

**Methods:**

ARTFORCE is a randomized phase II trial for 268 patients with a factorial 2 by 2 design: cisplatin versus Cetuximab and standard RT versus redistributed RT. Cisplatin is dosed weekly 40 mg/m^2^ for 6 weeks. Cetuximab is dosed 250mg/m^2^ weekly (loading dose 400 mg/m^2^) for 6 weeks. The standard RT regimen consists of elective RT up to 54.25 Gy with a simultaneous integrated boost (SIB) to 70 Gy in 35 fractions in 6 weeks. Redistributed adaptive RT consists of elective RT up to 54.25 Gy with a SIB between 64-80 Gy in 35 fractions in 6 weeks with redistributed dose to the gross tumour volume (GTV) and clinical target volume (CTV), and adaptation of treatment for anatomical changes in the third week of treatment.

Patients with locally advanced, biopsy confirmed squamous cell carcinoma of the oropharynx, oral cavity or hypopharynx are eligible.

Primary endpoints are: locoregional recurrence free survival at 2 years, correlation of the median ^89^Zr-cetuximab uptake and biological markers with treatment specific outcome, and toxicity. Secondary endpoints are quality of life, swallowing function preservation, progression free and overall survival.

**Discussion:**

The objective of the ARTFORCE Head and Neck trial is to determine the predictive value of biological markers and ^89^Zr-Cetuximab uptake, as it is unknown how to select patients for the appropriate concurrent agent. Also we will determine if adaptive RT and dose redistribution improve locoregional control without increasing toxicity.

ClinicalTrials.gov Identifier: NCT01504815

## Background

At diagnosis, 60% of the patients with head and neck cancer have locally advanced disease, requiring multi-modality treatment. For resectable disease, this treatment consists of surgery and radiotherapy (RT) with or without chemotherapy. For irresectable disease, the combination of radiotherapy and chemotherapy (chemoradiotherapy) is the treatment of choice. Chemoradiotherapy for head and neck cancer typically entails radiotherapy of the tumour and areas at risk for sub-clinical disease up to 70 Gy in combination with the chemotherapeutic agent cisplatin in different dose schemes. [1] Squamous cell carcinoma of the head and neck (SCCHN) unfortunately still has a poor prognosis, which is mainly due to failure of locoregional control. [1] Improving locoregional control for patients with locally advanced head and neck cancer is the objective of our study.

This study is part of a large European research project which investigates Adaptive and innovative Radiation Treatment FOR improving Cancer treatment outcomE (ARTFORCE). In this study, patients are randomized (a) between standard radiotherapy and adaptive radiotherapy combined with redistribution through dose-painting, and (b) between concurrent treatment with either Cetuximab or cisplatin. The following considerations have contributed to this concept.

**Figure 1:**
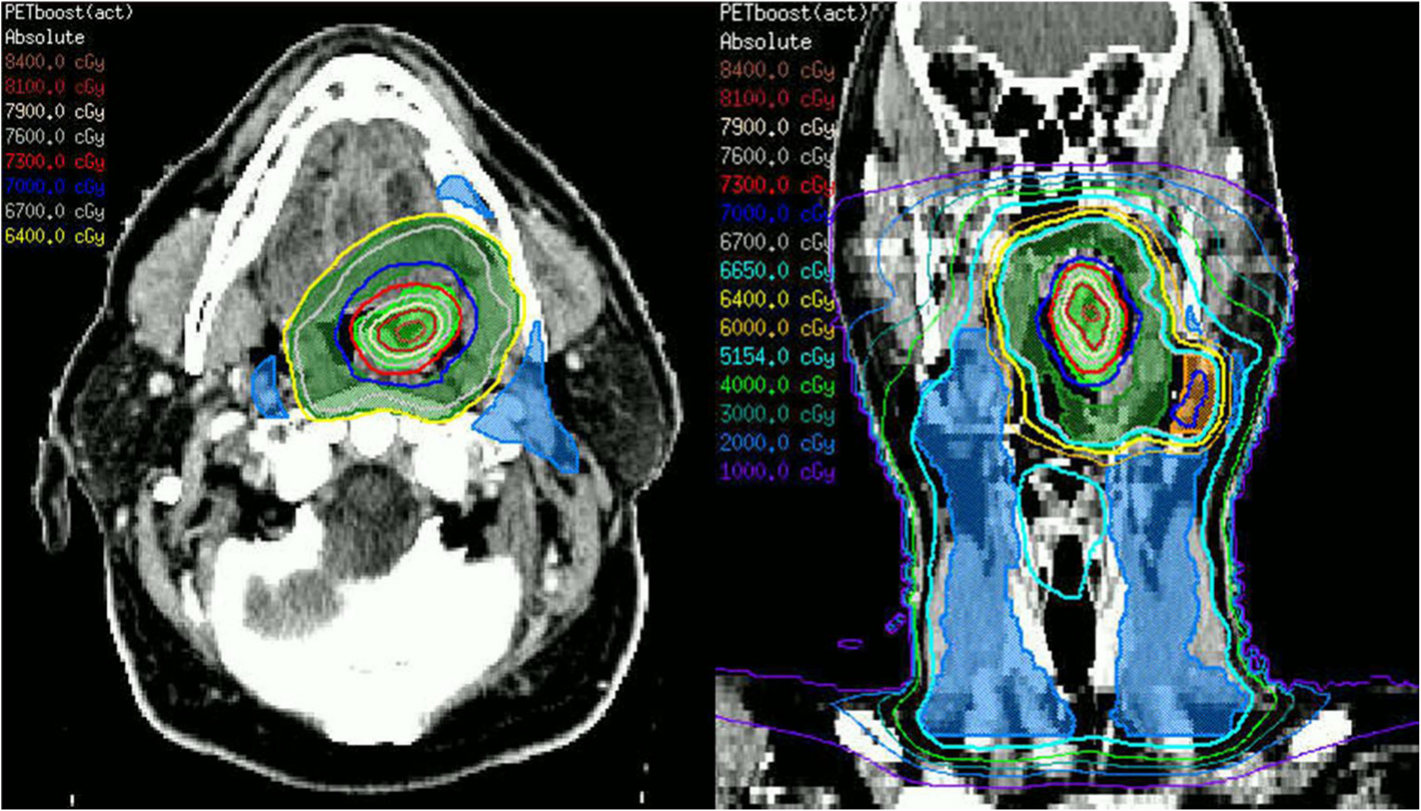
**Dose in the redistributed radiotherapy regimen.** Transverse (left) and coronal (right) view with isodose lines. In the dose-painted redistriburtion radiotherapy regimen, the GTV-FDG-PET is defined by an automatic iso-contour at 50% of the maximum uptake in the primary tumour. This is expanded by 3mm to form the PTV-FDG-PET. The GTV-primary should at least encompass the GTV-FDG-PET and is expanded by 10mm to form the PTV-primary. The PTV-FDG-PET will receive 35 fractions to a maximum total dose of 84 Gy to 2% of the volume (PTV-FDG-PET), a minimum dose of 70 Gy and a mean dose of approximately 77 Gy. The PTV-primary outside the PTV-FDG-PET will receive 35 fractions to a total mean dose of 67 Gy (ranging from 64-70 Gy).

Firstly, analyses of locoregional recurrence patterns show failure predominantly inside the gross tumour volume (GTV) of the primary tumour. [2] We therefore proposed to increase the dose to the most active part of the GTV primary as shown on F-18-fluorodeoxyglucose- positron emission tomography (FDG-PET) scan. [3] The dose is only increased in the primary tumour, because persistent neck nodes are salvable with surgery. Preliminary results indicate feasibility of focal dose escalation to the FDG-PET positive region, with acceptable toxicity. [4] Furthermore, adaptive replanning to account for anatomical changes that occur during the irradiation period effectively improves the sparing of organs at risk [5]. Hence, in the ARTFORCE trial, we will randomize patients for either the standard radiotherapy regimen (aiming to deliver a homogeneous dose of 70 Gy to the primary tumour) or a dose-painted - redistributed dose. In the dose-painted redistribution radiotherapy regimen, first the region of the primary tumour with at least 50% of its maximum FDG uptake is defined. Subsequently, a heterogeneous dose distribution is optimized aiming to deliver a maximum dose of 84 Gy to the high FDG uptake region and at least 64 Gy to the primary tumour (Figure 1). Adaptive re-planning occurs after the second week of treatment. This way, highly individualized radiotherapy can be prescribed.

Secondly, cisplatin is not the only possible agent suited for concurrent treatment. The use of Epidermal Growth Factor Receptor (EGFR) antibodies such as Cetuximab, for instance, is known to lead to similar results. [6,7] Even though the results of treatment with cisplatin and Cetuximab are comparable, it is not likely that these agents are equally effective in all patients, e.g. most patients will be cured by either treatment, yet some only by one. Treatment outcome might thus be improved by selecting the right drug for each patient. Currently, however, it is still unknown how to determine which treatment is optimal for which patient.

In this trial we evaluate the predictive value of biomarkers and pre-treatment in vivo uptake of Cetuximab in the tumour using zirconium-labelled (^89^Zr –labelled) Cetuximab on a PET scan [8,9].

To summarize: this article describes the ARTFORCE head & neck study protocol. ARTFORCE is a randomized multi-centre phase II trial with a factorial 2 by 2 design. In this study, standard radiotherapy is compared to redistributed adaptive radiotherapy with regard to locoregional control, disease free survival and toxicity. Frozen tumour biopsies and blood are collected for biomarkers and ^89^Zr-Cetuximab scans are performed to evaluate their predictive value in allocating patients for chemoradiotherapy with either concurrent Cetuximab or cisplatin.

### Study objectives and endpoints

Objectives:

•To determine if adaptive RT and dose redistribution improve locoregional control without increasing toxicity.

•To determine if ^89^Zr-Cetuximab uptake and biological markers predict treatment specific outcome.

Primary endpoints

a. Locoregional recurrence free survival at 2 years

b. Correlation of the median ^89^Zr-Cetuximab uptake and biological markers with treatment specific outcome

c. Toxicity

Secondary endpoints

d. Quality of life after treatment, at 6 months and 1 year

e. Swallowing function preservation at 1 year

f. Progression Free Survival

g. Overall Survival

## Methods

### Study design

**Figure 2:**
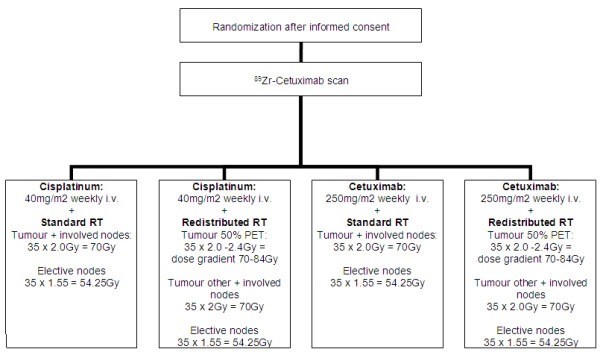
**The ARTFORCE head & neck study design.** (“tumour other” = tumour - 'tumour 50% isocontour PET’).

This study has a 2 by 2 design in which conventional versus redistributed radiotherapy are compared, as well as cisplatin versus Cetuximab (Figure 2). This leads to 4 different treatment arms. The first arm is considered the standard treatment: conventional radiotherapy with concomitant cisplatin. The second arm entails dose redistributed adaptive radiotherapy with concomitant cisplatin. Arm 3 and 4 both have Cetuximab regimens, the former combined with standard radiotherapy, the latter with redistributed adaptive radiotherapy.

### Sample size estimation

Sample size estimations are based on the comparison between conventional and redistributed radiotherapy. The study was designed to detect a 15% improvement in locoregional control with a power of 80% at a significance level of 0.05. The current locoregional recurrence rate at two years was estimated to be 35%. To achieve this significance level, 74 events need to be observed. Based on four years of accrual, and 1 year of follow up, 268 patients need to be included.

### In- and exclusion criteria

Prior to treatment, all patients are clinically evaluated by a multidisciplinary team consisting of at least a head and neck surgeon, radiation-oncologist and a medical-oncologist. Patients with histological proven stage III-IV, T3 - T4 squamous cell carcinoma of the head and neck are eligible for this trial and can be randomized after informed consent. Age limitations are set between 18 and 70 years. Patients need to be fit for chemoradiotherapy with a performance status 0–1 according to the World Health Organization (WHO) classification.

The main exclusion criteria are previous malignancies, prior treatment (surgery, chemotherapy or radiotherapy) for this tumour and/or active bacterial or viral infection.

### Pre-treatment evaluation

Standard pretreatment evaluation includes a FDG-PET-CT, magnetic resonance imaging (MRI) or CT scan of the head and neck area, ultrasound (US) of the neck including fine needle aspiration (FNAC) in case of suspicious nodes, electrocardiogram, audiometry and laboratory assessment. HPV status determination for oropharynx carcinoma is mandatory, as well as an investigation under general anesthesia with tumour biopsy. Extra for this study are the collection of blood samples and a frozen biopsy for biomarkers. Swallowing video fluoroscopy is optional.

Stratification will be done for tumour stage, tumour site (oral cavity vs oropharynx vs hypopharynx), primary tumour volume (< 30 cc vs 30 cc and above) and HPV-status for oropharynx carcinoma.

### Interventions

#### Pre-treatment ^89^Zr-Cetuximab scan

**Figure 3:**
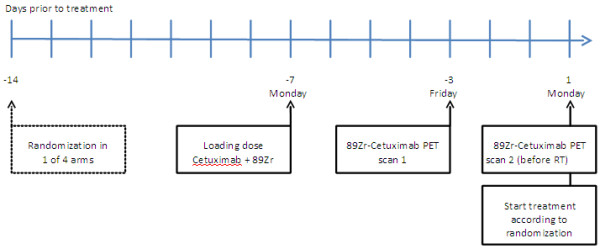
^**89**^**Zr-Cetuximab timeline.** Day −3 is three days before the first day of treatment. Between day −14 and −7, further pre-treatment evaluation is performed and the treatment planning is finalized.

**Figure 4:**
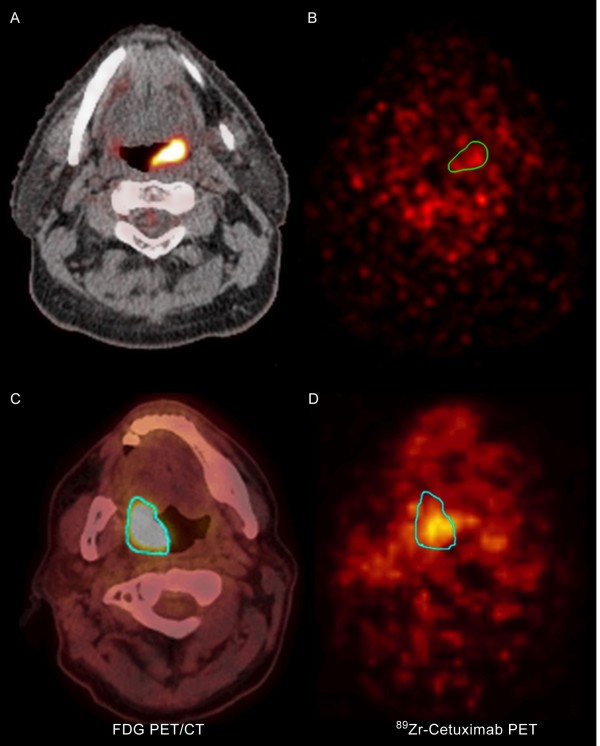
**Tumour imaging: FDG-PET/CT scans and **^**89**^**Zr-Cetuximab-PET scans. A** + **B**: patient with a negative ^89^Zr-Cetuximab scan. **C** + **D**: patient with a positive ^89^Zr-Cetuximab scan.

A week before the start of treatment a loading dose of Cetuximab 400mg/m^2^ will be administered and immediately followed by the intravenous (i.v.) administration of Zirconium labelled Cetuximab (^89^Zr-Cetuximab) (60mBq). The first 30 patients will be scanned twice to determine the optimal scanning moment to determine Cetuximab uptake in the tumour. (Figure 3 and Figure 4). These (^89^Zr-Cetuximab-PET) scans will be made 4 and 7 days after ^89^Zr-Cetuximab administration. For the rest of the study population we will select only one time moment for the ^89^Zr-Cetuximab-PET. The aortic arch will also be scanned and will serve as a reference point for ^89^Zr-Cetuximab uptake.

### Treatment: radiotherapy regimens

The clinical target volume (CTV) around the primary tumour is defined as the gross tumour volume (GTV) + 3D margin of 10mm, adjusted for anatomical borders in which microscopic disease is unlikely to extend. The CTV around the pathological lymph nodes is defined as the gross GTV + 3D margin of 5mm, adjusted for anatomical borders in which microscopic disease is unlikely to extend. Elective lymph node regions are defined in the protocol based on tumour site and stage, delineation should be done according to published guidelines [10,11]. Planning Target Volumes (PTV) are constructed by extension of the CTV with a 3D margin of 3-5mm according to hospital policy. The PTV will be irradiated with external beam radiotherapy using intensity modulated radiotherapy (IMRT) or Volumetric Modulated Arc Therapy (VMAT/ Rapid Arc) techniques. A simultaneous integrated boost (SIB) technique will be used. Six fractions per week will be given. The 6th fraction shall be given as a second fraction on one of the weekdays with an interval of at least 6 hours. Overall treatment time will be 39–40 days. Daily cone beam CT scans will be made during treatment to verify correct positioning of the patient before RT. Finally, daily Electronic Portal Imaging Device (EPID) dosimetry will be used for verification of actual radiation dose. Instructions on dose prescription and margins, as well as dose restrictions and delineation guidelines for organs at risk are available online at http://www.cancerartforce.eu.

#### Standard radiotherapy

The standard radiotherapy regimen consists of 35 fractions of 2.0 Gy to a total dose of 70 Gy to the PTVs of the primary tumour and the affected lymph nodes. The PTV elective lymph nodes will receive 35 fractions of 1.55 Gy to a total dose of 54.25 Gy.

#### Redistributed adaptive radiotherapy

In the dose-painted redistriburtion radiotherapy regimen, the GTV-FDG-PET is defined by an automatic iso-contour at 50% of the maximum uptake in the primary tumour. This is expanded by 3mm to form the PTV-FDG-PET. The GTV-primary should at least encompass the GTV-FDG-PET and is expanded by 10mm to form the PTV-primary, as described earlier.

The PTV-FDG-PET will receive 35 fractions to a maximum total dose of 84 Gy to 2% of the volume (PTV-FDG-PET), a minimum dose of 70 Gy and a mean dose of approximately 77 Gy. The PTV-primary outside the PTV-FDG-PET will receive 35 fractions to a total mean dose of 67 Gy (ranging from 64-70 Gy).

To assure accurate delivery of the redistributed radiation plan in arm 2 and 4, a repeat CT is made in week 2 of treatment. After recalculation and adaptation of the treatment plan on this CT, the patient will start with the new plan in week 3 of treatment. This way, we can account for anatomical changes thereby improving the accuracy of the dose delivery and sparing organs at risk.

### Treatment: concomitant strategies

#### Cisplatin treatment

Cisplatin treatment consists of weekly administration of cisplatin 40mg/m^2^ intravenously (i.v.) for 6 weeks. All cisplatin doses are accompanied by pre- and post-hydration with 2.5L of fluids according to the institutional standards of practice to minimize the risk of renal damage. Anti-emetic medication will be given according to hospital standards.

#### Cetuximab treatment

Cetuximab treatment (250mg/m^2^ i.v.) is given weekly for 6 weeks. An infusion of Cetuximab is always preceded by an antihistamine and corticosteroid to prevent allergic reactions.[12] Also, patients receiving Cetuximab treatment are prescribed doxycycline and pre-emptive topical treatment i.e. skin moisturizers, sunscreen and topical steroid to prevent skin toxicity according to Lacouture et al. [13].

## Results

### Assessment of toxicity

Toxicity is scored at least weekly according to the common toxicity criteria for adverse effects (CTCAE) version 4.0. [14] Electronic Case Report Forms will be used to document toxicity. Quality of life is scored using the Quality of life questionnaire (QLQ)-C30 (version 3.0) and the head and neck Cancer-specific QLQ-H&N35 and EuroQol-5D.

The study protocol has instructions (available online at http://www.cancerartforce.eu) on dose reductions and other measures to be undertaken in case of toxicity. It also defines when treatment should be discontinued.

### Follow up

Patients are examined weekly during treatment. Follow up starts directly after treatment and continues once weekly for 3 weeks. Thereafter, follow up visits are every 3 months for the first two years and twice annually until at least 5 years of follow-up.

### Response evaluation

The first evaluation is performed 12 weeks after the end of treatment. Evaluation will consist of physical examination, an FDG-PET, and either CT or MRI according to hospital standard. If recurrence is suspected, US with FNAC and examination under anesthesia with biopsy shall be performed. The response will be classified according to RECIST (Response Evaluation Criteria in Solid Tumours, version 1.1) [15].

## Discussion

### Ethics

The study will be conducted according to the ICH Harmonized Tripartite Guideline for Good Clinical Practice. It will be conducted in agreement with the declaration of Helsinki. The study has been approved by the Medical Ethical Committee of the Netherlands Cancer Institute - Antoni van Leeuwenhoek Hospital. Before the start in other centers, the protocol will be approved by local medical ethical committees.

All patients are given oral and written information about the study. They are given sufficient time to consider participating. Randomization will be done after informed consent has been signed.

### Quality assurance

Quality assurance in this trial is important and has the following aspects. Delineation and treatment planning guidelines are described in the protocol to ensure uniformity between centers. These guidelines were discussed with all participants and consensus was reached. Subsequently, a contouring and planning dummy run was performed by all participating centers, using a contouring atlas and specific treatment planning guidelines. Feedback was given after discussion in a group of trained specialists at Netherlands Cancer Institute.

Furthermore, quality of imaging was assured by a dummy run in all centers. For FDG-PET this included a dummy run of phantom imaging, signal quantification, and clinical image evaluation. For ^89^Zr-Cetuximab PET this included transport of the radiolabelled product, imaging and signal quantification evaluation. Verification of ^89^Zr-Cetuximab quantification as well as ^89^Zr-Cetuximab PET scans on phantoms were performed by the VU medical centre, a centre with extensive experience in radiolabelling of drugs and image quality assurance.

### Side studies

Tumour biopsies and blood samples from patients in the head and neck trial will be studied for validation of the response–predictive value of various biomarkers. For instance, the genomic signature of the tumour material will be investigated. Also, biomarkers such as the “Chung profile”, human papilloma virus (HPV) status, EGFR status and CD44 expression will be assessed. Furthermore, the accumulated dose based on daily CBCT scans and EPID dosimetry will be used in Tumour Control Probability (TCP) / Normal Tissue Complication Probability (NTCP) studies [16-22].

### Current status

The participating institutes include The Netherlands Cancer Institute in Amsterdam, The Netherlands, and Maastro Clinics in Maastricht, The Netherlands. Other consortium partners, for which patient accrual will start at a later phase include: Institut Gustave Roussy in Paris, France, Karolinska Hospital in Stockholm, Sweden, Christie Hospital in Manchester, United Kingdom, Val d’hebron Hopital in Barcelona, Spain, The first patient started treatment in September 2012. Patient accrual is expected to continue for 4 years.

## Abbreviations

ARTFORCE: Adaptive and innovative Radiation Treatment FOR improving Cancer treatment outcomE; CTCAE: Common toxicity criteria for adverse effects; CTV: Clinical target volume; EGFR: Epidermal growth factor receptor; EPID: Electronic portal imaging device; FDG-PET: On F-18-fluorodeoxyglucose- positron emission tomography; FNAC: Fine needle aspiration; GTV: Gross tumour volume; HPV: Human papilloma virus; iv: Intravenously; IMRT: Intensity modulated radiotherapy; NTCP: Normal tissue complication probability; MRI: Magnetic resonance imaging; PET: Positron emission tomography; PTV: Planning target volume; RECIST: Response evaluation criteria in solid tumours; RT: Radiotherapy; SCCHN: Squamous cell carcinoma of the head and neck; SIB: Simultaneous integrated boost; TCP: Tumour control probability; US: Ultrasound; VMAT: Volumetric modulated arc therapy; WHO: World health organization; 89Zr: Zirconium

## Competing interests

MERCK supplied the Cetuximab. MERCK had no influence on study design and was only contacted after the study protocol had been set. We declare there are no competing interests.

## Authors’ contributions

OH, FH, JG, NS, ED, HT are principal investigators for the ARTFORCE study at the various institutes and have as such contributed to drafting the original study protocol, without which this manuscript could not have been written. CR was the original PI for the European Union Grant application and designed the study, this position is now held by HB. PL was second author of the original study protocol. JH wrote this manuscript. OH, MV, MB and JJS helped to draft the manuscript. WV is responsible for the zirconium scans and edited the figures. All authors read and approved the final manuscript.

## Authors’ information

JG is professor and head of the department of radiotherapy at Val d’Hebron Hospital, Barcelona, Spain. MV is professor and head of the department of radiotherapy at The Netherlands Cancer Institute, Amsterdam, The Netherlands. MB is professor and head of the department of ear, nose and throat oncology and head and neck surgery at The Netherlands Cancer Institute, Amsterdam, The Netherlands. NS is professor and associate medical director at the Christie NHS foundation trust, Manchester, United Kingdom. PL is professor and head of the department of radiotherapy at Maastro Clinics, Maastricht, The Netherlands. HB is professor and former head of the department of radiotherapy at The Netherlands Cancer Institute, Amsterdam, The Netherlands. CR is professor and head of the department of radiotherapy at Amsterdam Medical Centre, Amsterdam, The Netherlands.

## Pre-publication history

The pre-publication history for this paper can be accessed here:

http://www.biomedcentral.com/1471-2407/13/84/prepub
